# Trends in the incidence and mortality of transitional cell carcinoma of the bladder for the last four decades in the USA: a SEER-based analysis

**DOI:** 10.1186/s12885-019-5267-3

**Published:** 2019-01-10

**Authors:** Muneer J. Al-Husseini, Ahmad Kunbaz, Anas M. Saad, João Vasco Santos, Sami Salahia, Marium Iqbal, Fares Alahdab

**Affiliations:** 1 Faculty of Medicine, Ain Shams University, Cairo, Egypt; 2 Cerrahpasa Medical Faculty, Istanbul University, Istanbul, Turkey; 3 Clinical Oncology Department, Faculty of Medicine, Damascus University, Damascus, Syria; 4 MEDCIDS – Department of Community Medicine, Information and Health Decision Sciences, Faculty of Medicine, University of Porto, Porto, Portugal; 5 CINTESIS – Centre for Health Technology and Services Research, Porto, Portugal; 6 Public Health Unit, AceS Grande Porto VIII – Espinho/Gaia, Porto, Portugal; 7 Batterjee Medical College, Jeddah, Saudi Arabia; 8 Mayo Evidence-based Practice Center, Mayo Clinic, Rochester, MN USA

**Keywords:** Bladder cancer, Transitional cell carcinoma, SEER, Incidence, Mortality

## Abstract

**Background:**

Transitional cell carcinoma (TCC) accounts for around 95% of bladder cancers and is the 4th most common cancer among men and the tenth most common in women, in the US. There is a constant need to clarify current TCC incidence and mortality rates among different population groups for better clinical practice guidelines. We aimed to describe the TCC incidence and incidence-based mortality by demographic and tumor-related characteristics over the last 40 years in the US.

**Methods:**

We obtained data from the SEER 18 registries to study TCC cases that were diagnosed between the years 1973 and 2014. We calculated incidence rates and incidence-based mortality rates in different demographic and tumor-related characteristics and expressed rates by 100,000 person-years. We then calculated the annual changes in incidence and incidence-based mortality rates and displayed them as annual percent changes (APCs).

**Results:**

There were 182,114 patients with TCC between 1973 and 2014 in the United States. Overall incidence rates of TCC increased 0.16% (95% CI, 0.02–0.30, *p* = .02) per year over the study period. However, the incidence declined significantly since 2007; (95%CI,-1.89- -0.77, *p* < .001), except among the elderly and African Americans, which increased significantly over the study period. Overall TCC mortality rates did not change over the study period. However, since 2000 it started to decrease significantly.

**Conclusion:**

TCC incidence and incidence-based mortality rates had been showing significant increases over the previous decades. However, significant declines in both incidence and incidence-based mortality rates have been observed over the recent years, except in some patients with certain racial groups. Improved understanding of the etiological and ecological factors of TCC could lead to further declines in incidence and incidence-based mortality rates.

**Electronic supplementary material:**

The online version of this article (10.1186/s12885-019-5267-3) contains supplementary material, which is available to authorized users.

## Introduction

Transitional cell carcinoma (TCC) is a malignant cancer that originates from the transitional epithelial cells of the urinary tract. It accounts for about 95% of bladder cancers (BC). The remaining 5% consists of squamous cell carcinoma, adenocarcinoma, and small cell carcinoma [1, 2]. For 2018, the American Cancer Society expected about 81,190 new cases of bladder cancer with 17,240 deaths [3]. Even with optimal treatment, BC recurs in more than half of cases and may progress to muscle-invasive BC in up to 20% of these patients [4]. TCC is the 4th most common cancers among males and the 10th most common among females in the US [3]. In addition, higher incidence of BC has been found in different studies with male to female incidence ratios varies between 2:1 to 3:1 [5–7].

Several environmental factors have already been associated with TCC. Cigarette smoking is the most well-established factor, being responsible for about 55% of all cases in the US [8]. Aromatic amines are the primary carcinogens of BC in smoking population [9]. Following smoking, occupational exposures to various carcinogens such as polycyclic aromatic hydrocarbons and chlorinated hydrocarbons are correlated with 20% of cases, especially in the industrial areas processing paint and dye [10–13]. High levels of arsenic in the drinking water have also been associated with an increased risk of tumor progression [14].

Furthermore, obesity has been shown to be a major risk factor contributing to TCC; as shown by a meta-analysis on 15 cohort studies that include more than 38,000 TCC patients, which investigated the possible correlation between cancer and obesity [15]. They showed a 4.2% increase in the incidence of BC for each 5 kg/m^3^ increase in weight among patients [15]. In fact, Bhaskaran et al. have already shown that obesity is related to 20% of the new cases of TCC in Britain [16]. These findings are consistent with another study emphasized the possible role of obesity in increasing the risk of bladder cancer by 28% [17]. Nevertheless, studies showed that socioeconomic differences in income and health care services might also be associated with an increased incidence and mortality rates of the tumor [18, 19].

Several genetic factors contribute to tumor’s development; as glutathione S-transferases (GSTs), which encode important enzymes in the process of carcinogens detoxification, showed to play a major role in bladder cancer occurrence [20]. In a meta-analysis that included 63 studies, they studied the possible association between GSTM1 or GSTT1 polymorphism and bladder cancer susceptibility [21]. They reported a strong correlation between single gene deletion ‘GSTM1 or GSTT1’ or double deletions of GSTM1/GSTT1 with a higher risk of bladder cancer, especially among Caucasians and Asians [21]. Moreover, evidence showed that higher risk of BC was found in patients with lower acetylation activity due to N-acetyltransferase 2 (NAT2) gene mutation [22]. On the other hand, multiple somatic mutations were detected in BC patients; as Gui et al. confirmed the presence of several mutated genes in TCC patients that vary according to the grade of disease [23]. In another study that performed a full genomic analysis on 99 patients with TCC, researchers identified several altered genes and mutations that contribute to the disease [24]. These findings highlight the potential role of genetic variations in the classification, diagnosis and the new approach treatment of bladder cancer.

This tumor is relatively common in the elderly, as the median age of diagnosis is 72 years old for men and 75 years old for women [19]. Additionally, TCC showed to express significant disparities among races [25]. Although the incidence of the tumor is 2 times higher in whites in comparison to African Americans, [26] the latter have a worse prognosis and higher tumor stages at presentation [25]. In fact, the mortality rates are significantly higher in African Americans, older patients, and females [27–29].

In this study, we sought to characterize the time trends and epidemiological profile of TCC. Using data provided by the National Cancer Institute’s Surveillance, Epidemiology, and End Results (SEER) program between 1973 and 2014 in the US, we investigated incidence rates and mortality rates in respect to some risk factors, i.e. state, race, age, and gender. This study aims to provide an overview about the epidemiology of BC in the recent 40 years, which may enlighten researchers with a better understanding for the reasons behind the current cancer incidence for a better clinical practice guideline.

## Methods

### Study design

We performed a retrospective cohort following the guidelines of the STROBE checklist (Strengthening the Reporting of Observational Studies in Epidemiology Statements) [30].

### Data source

We used the SEER*stat software to access the SEER 9 registries, that cover about 9.4% of the general US population from 1973 to 2014 [31, 32].

### Study population

We included patients older than twenty years who were diagnosed with TCC of the bladder between the years 1973 and 2014, and whose diagnosis was not only based on death certificates or autopsies. To select eligible cases that met our criteria, we considered the following SEER variables: ‘site recode ICD-O-3/WHO 2008: Urinary bladder’ and ‘Histology recode - broad groupings: 8120-8139 transitional cell papillomas and carcinomas’. We analyzed the following variables within included patients: sex of the patient, race, age at diagnosis (or age at death in case of incidence-based mortality calculation), state, stage at diagnosis of TCC (based on SEER historic stage A classification), and the specific site in the bladder.

### Outcomes

We calculated overall incidence rates of TCC of the bladder and incidence according to the previously mentioned variables. We also calculated overall incidence-based mortality rates of TCC of the bladder and incidence-based mortality according to the same variables. We expressed all rates by 100,000 person-years after adjusting to the general US population. The definition of incidence-based mortality (case fatality) was made as the number of deaths due to TCC among the number of TCC cases diagnosed over person-time at risk among the population in the SEER states [33–35]. All rates were calculated between 1973 and 2014 except for subgroups whose recording started later in the SEER program, i.e. Washington (since 1974) and Georgia (since 1975). To describe the change in incidence and mortality rates over years, we calculated the Annual Percentage Change (APC), which represents the average annual increases/decreases of these rates.

### Statistical analysis

SEER*stat was used to calculate incidence and incidendce-based mortality. We then used the joinpoint regression software that analyzes rates over time and to detect annual increases/decreases of these rates (APCs), then selects the best statistical model that achieves the least number of joinpoints and calculates *P* values using t tests [34–37]. P values were considered significant when less than .05. All statistical tests were two-sided.

## Results

### Baseline characteristics

Over the study period, 182,114 patients were diagnosed with transitional cell carcinoma of the bladder (TCC). Most patients were males (75.1%) and Caucasian (91.3%) and had a localized tumor (76.7%). The most common site in the bladder was the lateral wall (20.4%). However, in about 41.3% of cases, the specific site in the bladder was unknown. Table 1 shows characteristics of the included patients as well as the data by US state.

**Table 1:** Transitional cell carcinoma of the bladder incidence and incidence-based mortality rates (1973–2014)

characteristic	Incidence of bladder cancer		Incidence-based mortality of bladder cancer	
	Cases, No (%)^a^	Rate (95% CI)^b^	Deaths, No (%)^a^	Rate (95% CI)^b^
Overall	182,114 (100)	26.43 (26.55–26.31)	123,137(100)	18.68 (18.57–18.78)
Sex				
Male	136,897 (75.1)	47.21 (46.95–47.46)	92,733 (75.3)	37.70 (37.45–37.95)
Female	45,217 (24.8)	11.48 (11.38–11.59)	30,404 (24.6)	7.41 (7.33–7.50)
Race				
Caucasian	166,426 (91.3)	28.77 (28.63–28.91)	113,599 (92.2)	20.13 (20.02–20.25)
African American	7939 (4.3)	15.07 (14.73–15.41)	5493 (4.4)	11.92 (11.60–12.25)
Others^c^	6798 (3.7)	12.04 (11.75–12.34)	3866 (3.1)	7.72 (7.48–7.97)
Age at diagnosis, y				
20–44	5527 (3.0)	1.57 (1.53–1.62)	353 (0.4)	0.10 (0.09–0.12)
45–64	50,194 (27.5)	21.01 (20.83–21.2)	10,369 (8.42)	4.25 (4.17–4.33)
65–84	107,382 (58.9)	103.89 (103.27–104.51)	70,946 (57.6)	70.2 (69.68–70.72)
> 84	19,011 (10.4)	134.61 (132.7–136.53)	41,469 (33.6)	293.62 (290.8–296.46)
State				
California	26,322 (14.4)	24.07 (23.78–24.37)	18,017 (14.6)	17.21 (16.96–17.47)
Connecticut	33,844 (18.5)	32.3 (31.95–32.64)	22,862 (18.5)	21.99 (21.71–22.28)
Georgia	10,954 (6)	22.29 (21.86–22.72)	6824 (5.5)	16.16 (15.77–16.55)
Hawaii	5744 (3.2)	17.85 (17.39–18.32)	3650 (2.9)	11.99 (11.61–12.39)
Iowa	26,254 (14.4)	26.95 (26.62–27.28)	18,888 (15.3)	18.34 (18.08–18.61)
Michigan	33,036 (18.1)	29.51 (29.19–29.83)	22,985 (18.6)	21.98 (21.7–22.27)
New Mexico	8838 (4.9)	19.88 (19.47–20.31)	5912 (4.8)	14.63 (14.26–15.01)
Utah	8769 (4.8)	21.15 (20.71–21.60)	5689 (4.6)	15 (14.61–15.39)
Washington	28,353 (15.5)	29.13 (28.79–29.48)	18,310 (14.8)	19.95 (19.66–20.24)
Stage at diagnosis^d^				
Localized	139,766 (76.7)	20.24 (20.14–20.35)	87,384 (70.9)	13.32 (13.24–13.41)
Regional	31,612 (17.3)	4.62 (4.57–4.67)	26,214 (21.2)	3.93 (3.89–3.98)
Distant	5174 (2.8)	0.75 (0.73–0 .77)	4922 (3.9)	0.72 (0.70–0.74)
Site				
Trigone	10,849 (5.8)	1.57 (1.54–1.60)	7030 (5.7)	1.07 (1.04–1.09)
Dome	6176 (3.4)	0.91 (0.88–0.93)	4495 (3.7)	0.68 (0.66–0.70)
Lateral wall	37,061 (20.4)	5.35 (5.29–5.40)	23,266 (18.9)	3.53 (3.49–3.58)
Anterior wall	3112 (1.7)	0.46 (0.44–0.47)	2155 (1.8)	0.33 (0.31–0.34)
Posterior wall	14,482 (8)	2.11 (2.08–2.15)	9199 (7.5)	1.40 (1.37–1.43)
Neck	5478 (3.1)	0.8 (0.78–0.82)	3868 (3.1)	0.59 (0.57–0.61)
Ureteric orifice	9709 (5.3)	1.39 (1.36–1.42)	6451 (5.2)	0.98 (0.96–1)
Urachus	27 (0.01)	0.004 (0.003–0.006)	19 (0.01)	0.003 (0.002–0.005)
Overlapping lesions	19,999 (11)	2.91 (2.87–2.96)	15,138 (12.3)	2.29 (2.25–2.32)
Unknown	75,221 (41.3)	10.92 (10.84–11)	51,516 (41.8)	7.81 (7.74–7.88)

a Cases included first primary tumors that matched the selection criteria, were microscopically confirmed, and were not identified only from autopsy records or death certificates

b Rates were calculated as number of cases per 100,000 person-years and age adjusted to the 2000 US standard population

c Includes American Indian/Alaskan Native and Asian/Pacific Islander

d using SEER historic stage A

### Incidence rates and trends over time

Overall incidence of TCC during 1973–2014 was 26.43 (95% CI, 26.55–26.31]) per 100,000 person-years. TCC incidence was highest among males (47.21 [95% CI, 46.95–47.46]), Caucasians (28.77 [95% CI, 28.63–28.91]), and patients older than 84 years (134.61 [95% CI, 132.70–136.53]). When compared to states in the SEER 9 registries, incidence was the highest in Connecticut (32.30 [95% CI, 31.95–32.64]) and the lowest in Hawaii (17.85 [95% CI, 17.39–18.32]) (Table 1).

**Fig. 1:**
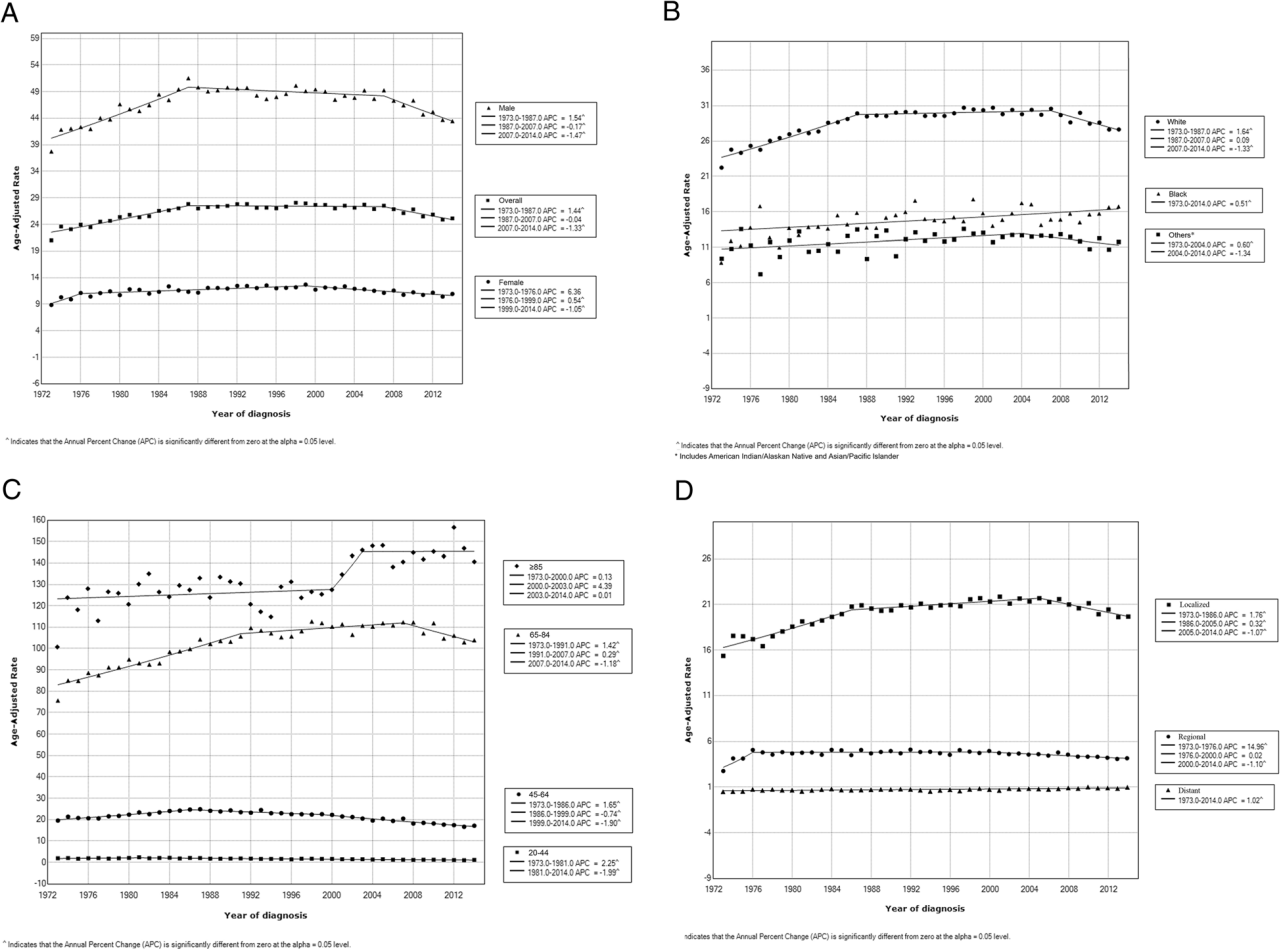
Trends in annual transitional cell carcinoma of the bladder incidence (1973–2014). **a** Overall transitional cell carcinoma of the bladder incidence trends, and incidence trends by sex. **b** Transitional cell carcinoma of the bladder incidence trends by race. **c** Transitional cell carcinoma of the bladder incidence trends by age. **d** Transitional cell carcinoma of the bladder incidence trends by stage

TCC incidence rates increased 0.16% (95% CI, 0.02–0.30, *p* = .02) per year over the study period, with an incidence rate of 21.02 in 1973 and of 25.13 in 2014 per 100,000 person-years. However, this increase was mainly during 1973–1987; APC, 1.45% (95% CI, 1.17–1.72, *p* < .001), then incidence rates became stable until 2007, when it started to decrease until 2014; APC, − 1.33% (95% CI, − 1.89 - -0.77, p < .001). During the recent years, the incidence of TCC has been decreasing in most subgroups. However, it has not changed recently among patients older than 84 years, and increased significantly among African Americans; APC, 0.60% (95% CI, 0.16–1.04, *p* = .01). Figure 1 and Table 2 describe TCC incidence trends during 1973–2014 by sex, race, age, and stage. This recent decline in incidence was also observed in some states (California, Georgia, Iowa, New Mexico, and Washington). Whereas the incidence has not been decreasing in other states. Additional file 1: Table S1 describes TCC incidence trends in detail during 1973–2014 by individual states.

**Table 2:** Trends in transitional cell carcinoma of the bladder Incidence Rates (1973–2014)

	Overall (1973–2014)		Trends								
			1			2			3		
	APC^a^ (95% CI)	P value^b^	year	APC^a^ (95% CI)	P value^b^	year	APC^a^ (95% CI)	P value^b^	year	APC^a^ (95% CI)	P value^b^
Overall	0.16 (0.02–0.30)	.02	1973–1987	1.45 (1.17–1.72)	<.001	1987–2007	−0.04 (− 0.17–0.10)	.61	2007–2014	− 1.33 (− 1.89 - -0.77)	<.001
Sex											
Male	0.02 (− 0.08–0.22)	.37	1973–1987	1.54 (1.24–1.85)	<.001	1987–2007	− 0.17 (− 0.31 - -0.02)	.03	2007–2014	− 1.47 (− 2.06 - -0.88)	<.001
Female	0.05 (− 0.12–0.22)	.56	1973–1976	6.36 (− 0.35–13.51)	.06	1976–1999	0.54 (0.31–0.78)	<.001	1999–2014	− 1.05 (− 1.40 - -0.70)	<.001
Race											
Caucasian	0.30 (0.15–0.44)	<.001	1973–1987	1.64 (1.36–1.91)	<.001	1987–2007	0.09 (− 0.05–0.23)	.19	2007–2014	− 1.33 (− 1.90 - -0.76)	<.001
African American	0.51 (0.27–0.75)	<.001	1973–2014	0.51 (0.27–0.75)	<.001						
Others^c^	0.13 (− 0.15–0.40)	.35	1973–2004	0.60 (0.16–1.04)	.01	2004–2014	− 1.34 (− 2.69–0.03)	.06			
Age at diagnosis, y											
20–44	− 1.61 (− 1.85 - -1.36)	<.001	1973–1981	2.25 (0.08–4.46)	.04	1981–2014	− 1.99 (− 2.22 - -1.76)	<.001			
45–64	−0.64 (− 0.86 - -0.41)	<.001	1973–1986	1.65 (1.24–2.06)	<.001	1986–1999	−0.74 (− 1.15 - -0.34)	.001	1999–2014	− 1.90 (− 2.16 - -1.64)	<.001
65–84	0.53 (0.38–0.67)	<.001	1973–1991	1.42 (1.17–1.66)	<.001	1991–2007	0.29 (0.04–0.54)	.03	2007–2014	− 1.18 (− 1.91 - -0.44)	.003
> 84	0.56(0.41–0.70)	<.001	1973–2000	0.13 (− 0.15–0.41)	.35	2000–2003	4.39 (− 7.05–17.23)	.46	2003–2014	0.01 (− 0.67–0.70)	.98
Stage at diagnosis^d^											
Localized	0.37 (0.22–0.52)	<.001	1973–1986	1.76 (1.38–2.13)	<.001	1986–2005	0.32 (0.15–0.50)	.001	2005–2014	− 1.07 (− 1.52 - -0.63)	<.001
Regional	−0.18 (− 0.39–0.02)	.08	1973–1976	14.96 (5.51–25.25)	.002	1976–2000	0.02 (− 0.24–0.29)	.86	2000–2014	− 1.10 (− 1.57 - -0.61)	<.001
Distant	1.02 (0.72–1.31)	<.001	1973–2014	1.02 (0.72–1.31)	<.001						

a Annual Percentage Changes, calculated using Joinpoint regression software

b Two-sided *P* value was calculated using t test to determine the significance of APC change

c Includes American Indian/Alaskan Native and Asian/Pacific Islander

d using SEER historic stage A

### Incidence-based mortality rates and trends over times

Overall incidence-based mortality rate of TCC during 1973–2014 was 18.68 [95% CI, 18.57–18.78]) per 100,000 person-years. TCC incidence-based mortality was highest among males (37.70 [95% CI, 37.45–37.95]), Caucasians (20.13 [95% CI, 20.02–20.25]), and patients older than 84 years (293.62, 95% CI, 290.80–296.46]). When compared to states in the SEER 9 registries, incidence-based mortality was highest in Connecticut (21.99 [95% CI, 21.71–22.28]), and lowest in Hawaii (11.99 [95% CI, 11.61–12.39]) (Table 1).

**Fig. 2:**
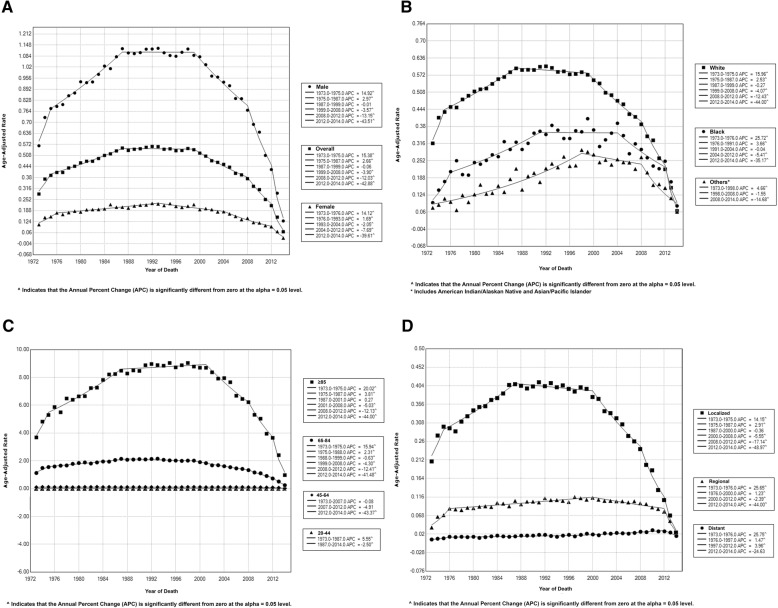
Trends in annual transitional cell carcinoma of the bladder incidence- based mortality (1973–2014). **a** Overall transitional cell carcinoma incidence-based mortality trends, and incidence-based mortality trends by sex. **b** Transitional cell carcinoma incidence-based mortality trends by race. **c** Transitional cell carcinoma incidence-based mortality trends by age. **d** Transitional cell carcinoma of the bladder incidence-based mortality trends by stage

Over the study period, TCC overall incidence-based mortality rates did not change significantly, with a rate of 0.28 in 1973 and of 0.07 in 2014, per 100,000 person-years. However, it started to decrease significantly after 2000, and this decrease continued to reach − 42.88% (95% CI, − 49.35 - -35.59, *P* < .001) between 2012 and 2014. This decline in incidence-based mortality since 2000 was observed in almost all subgroups, except for cases with distant metastasis in which mortality continued to increase until 2012 and did not change significantly then. Figure 2 and Table 3 describe TCC incidence-based mortality trends during 1973–2014 by sex, race, age, and stage. Mortality from bladder cancer has also been decreasing in most states since the 2000s. The only exception was New Mexico, where mortality decreased significantly between 2000 and 2012; APC, − 5.93% (95% CI, − 8.10 - -3.71, *P* < .001), but did not change significantly between 2012 and 2014. Additional file 2: Table S2 describes TCC incidence-based mortality trends during 1973–2014 by states.

**Table 3:** Trends in transitional cell carcinoma of the bladder Incidence-based mortality Rates (1973–2014)

	Overall (1973–2014)		Trends								
	APC^a^ (95% CI)	*P* value^b^	year	APC^a^ (95% CI)	*P* value^b^	year	APC^a^ (95% CI)	*P* value^b^	year	APC^a^ (95% CI)	*P* value^b^
Overall	−0.57 (− 1.27–0.14)	.12	1973–1975	15.38 (8.07–23.20)	<.001	1975–1987	2.66 (2.28–3.04)	<.001	1987–1999	−0.06 (− 0.39–0.27)	.72
			1999–2008	− 3.90 (− 4.48 - -3.31)	<.001	2008–2012	− 12.03 (− 14.94 - -9.02)	<.001	2012–2014	− 42.88 (− 49.35 - -35.59)	<.001
Sex											
Male	−0.45 (− 1.18–0.29)	.22	1973–1975	14.93 (6.75–23.73)	.001	1975–1987	2.97 (2.53–3.40)	<.001	1987–1999	− 0.01 (− 0.40–0.39)	.96
			1999–2008	− 3.57 (− 4.23 - -2.91)	<.001	2008–2012	− 13.15 (− 16.55 - -9.61)	<.001	2012–2014	− 43.51 (− 50.84 - -35.09)	<.001
Female	−0.68 (− 1.38–0.02)	.06	1973–1976	14.12 (5.50–23.45)	.002	1976–1993	1.69 (1.18–2.19)	<.001	1993–2004	− 2.05 (− 3.02 - -1.07)	<.001
			2004–2012	− 7.70 (− 9.55 - -5.80)	<.001	2012–2014	− 39.61 (− 55.68 - -17.72)	.002			
Race											
Caucasians	− 0.74 (− 1.47–0.001)	.05	1973–1975	15.96 (8.94–23.44)	<.001	1975–1987	2.53 (2.17–2.90)	<.001	1987–1999	− 0.27 (− 0.59–0.06)	.10
			1999–2008	− 4.07 (− 4.65 - -3.48)	<.001	2008–2012	− 12.43 (− 15.50 - -9.25)	<.001	2012–2014	− 44.00 (− 50.11 - -37.14)	<.001
African Americans	0.68 (− 0.10–1.46)	.09	1973–1976	25.72 (6.28–48.70)	.01	1976–1991	3.66 (2.50–4.83)	<.001	1991–2004	− 0.04 (− 1.30–1.23)	.95
			2004–2012	− 5.41 (− 8.38 - -2.34)	.001	2012–2014	− 35.17 (− 54.87–6.88)	.02			
Others^c^	1.68 (0.77–2.60)	.001	1973–1998	4.66 (3.83–5.50)	<.001	1998–2008	−1.55 (− 4.26–1.24)	.26	2008–2014	−14.68 (− 20.74 - -8.17)	<.001
Age at death, y											
20–44	−0.61 (−1.60–0.40)	.23	1973–1987	5.55 (1.54–9.73)	.01	1987–2014	− 2.50 (− 3.91 - -1.08)	.001			
45–64	− 0.52 (− 0.91 - -0.13)	.01	1973–2007	−0.08 (− 0.34–0.17)	.51	2007–2012	−4.91 (− 11.30–1.94)	.15	2012–2014	− 43.37 (− 63.12 - -13.06)	.01
65–84	− 0.85 (− 1.55 - -0.14)	.02	1973–1975	15.94 (7.25–25.34)	.001	1975–1988	2.31 (1.91–2.70)	<.001	1988–1999	− 0.63 (− 1.11 - -0.14)	.01
			1999–2008	− 4.30 (− 5.02 - -3.57)	<.001	2008–2012	− 12.41 (− 16.18 - -8.47)	<.001	2012–2014	− 41.48 (− 50.01 - -31.50)	<.001
> 84	−0.10 (− 0.90–0.71)	.81	1973–1975	20.02 (5.72–36.27)	.01	1975–1987	3.81 (3.12–4.50)	<.001	1987–2001	0.27 (− 0.19–0.74)	.24
			2001–2008	− 5.03 (− 6.56 - -3.48)	<.001	2008–2012	− 12.13 (− 17.30 - -6.62)	<.001	2012–2014	− 44.00 (− 55.10 - -30.16)	<.001
Stage at diagnosis^d^											
Localized	−0.79 (−1.60–0.03)	.06	1973–1975	14.15 (4.78–24.37)	.004	1975–1987	2.91 (2.41–3.42)	<.001	1987–2000	−0.36 (− 0.75–0.04)	.08
			2000–2008	−5.55 (− 6.48 - -4.62)	<.001	2008–2012	−17.14 (− 21.68 - -12.35)	<.001	2012–2014	−48.97 (− 59.18 - -36.19)	<.001
Regional	0.43 (− 0.16–1.03)	.15	1973–1976	25.65 (14.69–37.67)	<.001	1976–2000	1.23 (0.88–1.58)	<.001	2000–2012	− 2.39 (− 3.39 - -1.38)	<.001
			2012–2014	− 44.00 (− 57.08 - -26.94)	<.001						
Distant	2.43 (2.06–2.80)	<.001	1973–1976	25.75 (1.88–55.20)	.03	1976–1997	1.47 (0.67–2.27)	.001	1997–2012	3.97 (2.66–5.29)	<.001
			2012–2014	−24.63 (− 46.37–5.93)	.10						

a Annual Percentage Changes, calculated using Joinpoint regression software

b Two-sided P value was calculated using t test to determine the significance of APC change

c Includes American Indian/Alaskan Native and Asian/Pacific Islander

d using SEER historic stage A

## Discussion

The present comprehensive study of TCC trends over the past 40 years show an overall increase in incidence, though a contrary trend was verified since 2007 accompanied with an overall decrease mortality rates since 2000 in all groups except distant stage cancer patients in the US. More specifically, the increased incidence is significant among African Americans and the most diagnosed tumors were in the localized stage (76.6%). The incidence of both localized and regional tumor decreased significantly in recent years. Nevertheless, most cases of TCC present in men and Caucasians that have the highest mortality rates.

Our study showed an overall increase in the incidence of TCC in all groups. However, recent years showed a decline in all groups except for African Americans. Similarly, earlier studies determined the same results with an increased incidence of localized stage cancer except for African Americans where the increase was detected in all BC stage groups [38, 39]. The overall increase in incidence could be partially explained by the significant use of imaging techniques such as ultrasonography, computed tomography and magnetic resonance image within the same period as diagnostic tools [40, 41]. In addition, different biomarkers are currently used for the early detection of BC [42–44]. But, the increased incidence among African Americans in all BC stages indicates the incapability of these advanced diagnostic methods alone to illustrate the rapidly increasing incidence among different racial groups. [39] Although SEER database represents only 10% of the US population, it has provided researchers with a valid epidemiological tool to investigate different cancers prevalence in the US and therefore the results may vary according to the available data.

A study about the trends and patterns of BC incidence, which analyzed data from the International Agency for Research on Cancer and the World Health Organization, showed a strong correlation between BC incidence and tobacco smoking prevalence worldwide [45]. According to our results, the incidence of BC dropped significantly since 2008 for most of the study groups. These declines may be correlated with a recent report from the Centers for Disease Control and Prevention’s (CDC), demonstrating the significant drop in smoking trends among adults from 42.4% in 1965 to 16.8% in 2014 in the US [46]. Brennan et al. showed a significant correlation between cancer incidence with a longer duration of smoking and higher daily smoking habits [47].

It was estimated that 50% of BC cases in males and 25% of BC cases in females could be eliminated by smoking cessation [48]. Moreover, smoking cessation showed an immediate decrease in BC risks [47]. Cigarette smoking showed a significant association with increased risk of both low-grade and invasive bladder cancer [49]. Interestingly, smoking showed to not only be associated with higher incidence of TCC but also a higher grade of tumor at presentation and worse prognosis [50]. In contrast, a recent study showed a weak increase in the risk of a more aggressive tumor type with increasing smoking intensity, indicating the needs for more studies to clarify the results [51].

A recent data suggested the role of socioeconomic factors based on agricultural, industrial and residential land use on the BC occurrence [19]. To clarify this point, a study about trends of BC incidence in Asian countries showed that developed countries had a higher incidence, but better survival rates in comparison to the developing countries [52]. Another study in Europe investigated the trend of BC between 1970 and 2008 confirmed the latter findings as the western countries showed to have more favorable BC mortality rates in comparison to some eastern countries [53]. In addition, early reports indicated that smoking rates could be related to the income and employment status [54, 55]. These findings suggest the need for further epidemiological study of potential mechanisms of BC occurrence [56].

Our results show the dominance of TCC cases in men. Disparities among gender showed to be associated with different epidemiological and genetical factors such as smoking, occupational risk factors, tumor biology, and different sex hormones mechanisms [57]. Hemelt et al. showed unexpected higher male to female ratios, but with low smoking prevalence in female “10%” in comparison to males “75%”, concluding that these differences among gender cannot be explained only by the high smoking prevalence in males [58]. In addition, recent evidence indicated the important role of certain enzymatic isoforms in the liver ‘the primary site for TCC carcinogens metabolism’, resulting in different exposure risk of the urothelium to carcinogens, which as a result may contribute to the gender differences of TCC incidence [59]. Moreover, estrogen showed to inhibit bladder carcinogenesis during the progression phases, which showed to be promoted in the presence of androgens. However, the mechanisms underlying these findings need further investigations [59]. These findings were consistent with the results of Davis-Dao et al. that indicated a 30% lower risk of BC in parous women comparing to nulliparous women [60]. Another nationwide population-based cohort indicated the higher incidence of BC in uniparous women compared to those who had more than one child, [61] Supporting the evidence regarding the protective properties of estrogen against TCC [59]. Gender differences seem to be related to many potential biologic and epidemiologic factors that contributed to the disparity in incidence, stage at presentation and outcomes [62].

However, as aging is considered as a high-risk factor for tumors incidence, persons over 65 accounts for 60% of newly diagnosed cancers and 70% of all malignancies deaths [63]. In our study, these patients accounted for 58.9% of the cases. Furthermore, in a recent study investigated trends for the stage-specific incidence of BC between 1988 and 2006, they found a dramatic increase of BC among elderly populations [56]. Researchers suggested the lack of sufficient clinical data guiding treatment decisions in older patients as a main contributor to the increased incidence among elderlies [64].

Mortality represents a more accurate measurement of cancer control outcomes than survival rates [65]. Since 2000, we found significant declines in mortality rates for all groups. Supporting our findings, a positive trend toward lower mortality rates have been detected in the US, Europe and around the world [45, 66, 67]. Between 1975 and 1995 and 1996–2009, several studies indicated an increase in the 5-year survival rates for localized, regional stages tumor while the survival of distant stage remained stable [38, 39]. The moderate decrease may possibly be due to better diagnosis and utilization of traditional treatment methods. A part of the decrease in mortality might be due to the lower smoking prevalence in the US in the last decade [68]. Other factors such as socioeconomic status may contribute to the overall mortality rates [69]. In our study, the mortality rates among African Americans slightly decreased (not significantly) over the last few years. Although this disparity does not appear to be due to differences in the intensity or quality of care provided, [70] African Americans with TCC have a higher stage at presentation, more unfavorable histology findings, and poorer survival in comparison to Caucasians [28, 71, 72]. However, in our study, we reported a higher mortality rate among Caucasians in comparison to African Americans, which could be partially explained by the fact that SEER database represents only 10% of the total US population. Overall, the heterogeneity of the tumor behavior indicates the lack of effective treatment modalities that could reduce the mortality rates significantly [73, 74].

The current study may have limitations related to the classification of tumor stages (Tis, Ta, T1, and T2) due to the nature of data presented in SEER. Consequently, we analyzed all stages in one group as a localized stage. In addition, we were unable to analyze the different prognostic factors that showed roles in the incidence of TCC using the currently available data. These limitations may reduce our understanding of the correlations among these factors with TCC.

## Conclusions

Overall, our study showed a recent decrease in the incidence of TCC in different age, sex or race groups. We also indicated the time trends and incidence of slightly decreased mortality rates, which could be linked to the recent advances in treatment. More efforts are needed to increase the survival of these patients. Ecological studies might be performed in order to study correlations between demographic and socioeconomic status and incidence and mortality of this disease. Therefore, our study raised some questions about the etiological factors that may contribute to the incidence of increment over the last four decades, which as a result require further investigation.

## Additional files

Additional file 1: **Table S1.** Trends in transitional cell carcinoma of the bladder Incidence Rates by state (1973–2014). (DOCX 20 kb)

Additional file 2: **Table S2.** Trends in transitional cell carcinoma of the bladder Incidence-based mortality Rates by state (1973–2014). (DOCX 24 kb)

## Abbreviations

APCs: Annual Percentage Changes
BC: Bladder cancers
CDC: The Centers for Disease Control and Prevention
GSTs: Glutathione S-transferases
NAT2: N-acetyltransferase 2
SEER: Surveillance, Epidemiology, and End Results program
TCC: Transitional cell carcinoma
