# Supplementary material for: Trends in the incidence and mortality of transitional cell carcinoma of the bladder for the last four decades in the USA: a SEER-based analysis
Source: BMC Cancer. 2019 Jan 10;19:46. doi: 10.1186/s12885-019-5267-3 (PMC6327491; doi:10.1186/s12885-019-5267-3)
Supplement: Supplementary file 1 — Table S1. Trends in transitional cell carcinoma of the bladder Incidence Rates by state (1973–2014). (DOCX 20 kb) [file 12885_2019_5267_MOESM1_ESM.docx]

**Additional file 1: Table S1** Trends in transitional cell carcinoma of the bladder Incidence Rates by state (1973-2014)

|  | Overall  (1973-2014)^a^ | | Trends | | | | | | | | |  |
| --- | --- | --- | --- | --- | --- | --- | --- | --- | --- | --- | --- | --- |
|  |  |  | 1 | | | 2 | | | 3 | | | |
|  | APC^b^  (95% CI) | P value^c^ | year | APC^b^  (95% CI) | P value^c^ | year | APC^b^  (95% CI) | P value^c^ | year | APC^b^  (95% CI) | P value^c^ | |
| California | -0.23  (0.37 - -0.09) | .002 | 1973-1991 | 0.55  (0.15 - 0.96) | .01 | 1991-2014 | -0.7  (-0.94 - -0.46) | <.001 |  |  |  | |
| Connecticut | 0.59  (0.44 - 0.74) | <.001 | 1973-1992 | 1.54  (1.22 - 1.86) | <.001 | 1992-2014 | -0.01  (-0.22 - 0.19) | .91 |  |  |  | |
| Georgia | 0.01  (-0.3 - 0.33) | .95 | 1975-1992 | 1.75  (0.7 - 2.82) | .002 | 1992-2014 | -0.83  (-1.33 - -0.33) | .002 |  |  |  | |
| Hawaii | 0.13  (-0.17 - 0.44) | .39 | 1973-2014 | 0.13  (-0.17 - 0.44) | .39 |  |  |  |  |  |  | |
| Iowa | 0.43  (0.27 - 0.59) | <.001 | 1973-1985 | 1.76  (1.01 - 2.51) | <.001 | 1985-2009 | 0.3  (0.06 - 0.54) | .02 | 2009-2014 | -2.43  (-4.67 - -0.13) | .04 | |
| Michigan | 0.50  (0.31 - 0.68) | <.001 | 1973-1980 | 3.63  (1.59 - 5.71) | .001 | 1980-2000 | 0.66  (0.30 - 1.02) | .001 | 2000-2014 | -0.48  (-0.99 - 0.04) | .07 | |
| New Mexico | -0.06  (-0.4 - 0.28) | .72 | 1973-1988 | 3.02  (1.66 - 4.4) | <.001 | 1988-2014 | -0.91  (-1.3 - -0.52) | <.001 |  |  |  | |
| Utah | 0.13  (-0.06 - 0.32) | .16 | 1973-2014 | 0.13  (-0.06 - 0.32) | .16 |  |  |  |  |  |  | |
| Washington | 0.09  (-0.09 - 0.27) | .32 | 1974-2001 | 0.66  (0.42 - 0.9) | <.001 | 1991-2014 | -1.19  (-1.72 - -0.66) | <.001 |  |  |  | |

a Overall APC was calculated between 1973-2014 for all states except Georgia; 1975-2014, and Washington; 1974-2014

b Annual Percentage Changes, calculated using Joinpoint regression software

c Two-sided P value was calculated using t test to determine the significance of APC change
