# Supplementary material for: Trends in the incidence and mortality of transitional cell carcinoma of the bladder for the last four decades in the USA: a SEER-based analysis
Source: BMC Cancer. 2019 Jan 10;19:46. doi: 10.1186/s12885-019-5267-3 (PMC6327491; doi:10.1186/s12885-019-5267-3)
Supplement: Supplementary file 2 — Table S2. Trends in transitional cell carcinoma of the bladder Incidence-based mortality Rates by state (1973–2014). (DOCX 24 kb) [file 12885_2019_5267_MOESM2_ESM.docx]

Additional file 2: **Table S2** Trends in transitional cell carcinoma of the bladder Incidence-based mortality Rates by state (1973-2014)

|  | Overall  (1973-2014)^a^ | | Trends | | | | | | | | | | | |
| --- | --- | --- | --- | --- | --- | --- | --- | --- | --- | --- | --- | --- | --- | --- |
|  |  |  | 1 | | | 2 | | | 3 | | | 4 | | |
|  | APC^b^  (95% CI) | P value^c^ | year | APC^b^  (95% CI) | P value^c^ | year | APC^b^  (95% CI) | P value^c^ | year | APC^b^  (95% CI) | P value^c^ | year | APC^b^  (95% CI) | P value^c^ |
| California | -1.29  (-1.93 - -0.63) | <.001 | 1973-1991 | 1.52  (1.07 - 1.97) | <.001 | 1991-2007 | -2.68  (-3.27 - -2.08) | <.001 | 2007-2012 | -12.24  (-17.41 - -6.75) | <.001 | 2012-2014 | -48.04  (-64.11 - -24.76) | .001 |
| Connecticut | -0.68  (-1.36 - 0.02) | .06 | 1973-1986 | 3.06  (2.19 - 3.95) | <.001 | 1986-1999 | -0.05  (-0.92 - 0.83) | .91 | 1999-2010 | -4.51  (-5.75 - -3.25) | <.001 | 2010-2014 | -26.29  (-33.38 - -18.46) | <.001 |
| Georgia | -0.19  (-1.06 - 0.69) | .67 | 1975-1993 | 4.02  (3.09 - 4.96) | <.001 | 1993-2010 | -2.90  (-3.89 - -1.91) | <.001 | 2010-2014 | -25.45  (-35.73 - -13.53) | <.001 |  |  |  |
| Hawaii | 0.56  (-0.26 - 1.39) | .18 | 1973-1993 | 3.81  (2.59 - 5.04) | <.001 | 1993-2009 | -1.26  (-2.81 - 0.31) | .11 | 2009-2014 | -20.82  (-30.7 - -9.55) | .001 |  |  |  |
| Iowa | -1.14  (-1.77 - -0.51) | .001 | 1973-1989 | 1.84  (1.37 - 2.32) | <.001 | 1989-2006 | -1.95  (-2.4 - -1.49) | <.001 | 2006-2012 | -10.72  (-13.85 - -7.48) | <.001 | 2012-2014 | -47.86  (-62.17 - -28.15) | <.001 |
| Michigan | -0.88  (-1.58 - -0.19) | .01 | 1973-1984 | 3.16  (2.01 - 4.32) | <.001 | 1984-2000 | 0.05  (-0.59 - 0.70) | .87 | 2000-2012 | -6.45  (-7.64 - -5.25) | <.001 | 2012-2014 | -48.19  (-64.86 - -23.60) | .002 |
| New Mexico | 0.45  (-0.46 - 1.38) | .33 | 1973-1987 | 6.41  (4.78 - 8.06) | <.001 | 1987-2001 | 0.65  (-0.67 - 2) | .32 | 2000-2012 | -5.93  (-8.1 - -3.71) | <.001 | 2012-2014 | -39.2  (-63.49 - 1.28) | .056 |
| Utah | 0.34  (-0.35 - 1.04) | .32 | 1973-1999 | 2.24  (1.73 - 2.75) | <.001 | 1999-2011 | -3.31  (-4.95 - -1.65) | <.001 | 2011-2014 | -34.15  (-46.97 - -18.25) | <.001 |  |  |  |
| Washington | -0.27  (-1.02 - 0.48) | .47 | 1974-1987 | 3.66  (2.87 - 4.45) | <.001 | 1987-2002 | 0.29  (-0.32 - 0.9) | .34 | 2002-2011 | -6.26  (-7.75 - -4.76) | <.001 | 2011-2014 | -30.83  (-39.15 - -21.37) | <.001 |

a Overall APC was calculated between 1973-2014 for all states except Georgia; 1975-2014, and Washington; 1974-2014

b Annual Percentage Changes, calculated using Joinpoint regression software

c Two-sided P value was calculated using t test to determine the significance of APC change
